# Constraint Violations in Stochastically Generated Data: Detection and Correction Strategies

**DOI:** 10.1155/2014/370656

**Published:** 2014-02-04

**Authors:** Adam Fadlalla, Toshinori Munakata

**Affiliations:** ^1^Department of Accounting and Information Systems, Qatar University, P.O. Box 2713, Doha, Qatar; ^2^Department of Computer and Information Science, Cleveland State University, Cleveland, OH 44114, USA

## Abstract

We consider the generation of stochastic data under constraints where the constraints can be expressed in terms of different parameter sets. Obviously, the constraints and the generated data must remain the same over each parameter set. Otherwise, the parameters and/or the generated data would be inconsistent. We consider how to avoid or detect and then correct such inconsistencies under three proposed classifications: (1) data versus characteristic parameters, (2) macro- versus microconstraint scopes, and (3) intra- versus intervariable relationships. We propose several strategies and a heuristic for generating consistent stochastic data. Experimental results show that these strategies and heuristic generate more consistent data than the traditional discard-and-replace methods. Since generating stochastic data under constraints is a very common practice in many areas, the proposed strategies may have wide-ranging applicability.

## 1. Introduction

The use of stochastically (randomly) generated data is very common in various domains and for various reasons including:

- the Monte Carlo method where events (samples and data) are simulated by random numbers,
- verification of complex analytical solutions,
- assessment of heuristic methods through randomly generated test data,
- the so-called guided random search techniques, such as genetic algorithms and neural networks [1], which are particularly suited for search and optimization problems.

In these applications, certain probability distributions are assumed when generating random data. The distribution can be static or dynamic, depending on whether its associated parameters are fixed or are changing over time. Usually the generated data has to satisfy constraints in addition to the probability distribution constraints. All these constraints can be formulated in terms of different parameter sets. Each parameter set formulation must equivalently represent the same constraints.

We consider a problem where *n* events are randomly generated and each event is represented by *k* variables, *x*_1_,…, *x*_*k*_. For a simple queuing problem, *n* can represent the number of units to arrive at a service station, and *x*_1_ and *x*_2_ are service time and arrival time for each unit, respectively. Generally, the *n* samples can be represented by a *k* × *n* matrix as follows: *X* = [*x*_*ij*_], *i* = 1, *k*, *j* = 1, *n*, where *x*_*ij*_ represents the value of variable *x*_*i*_ for the *j*th sample. We note that the *i*th row of matrix *X* represents *n* samples of variable *x*_*i*_ and the *j*th column represents the values of *k* variables, *x*_1_,…, *x*_*k*_ for the *j*th sample. In this paper each *x*_*ij*_ is assumed as a scalar, for example, a real number between 0 and 1, or an integer in a certain range, say, between 0 and 10, depending on problem specifics. We generate *x*_*ij*_ randomly under a certain probability distribution and certain constraints. When we extend the assumption of each *x*_*ij*_ being a scalar, for example, to a vector, we would consider a scalar element of the vector.

Let the set of constraints to be imposed on *X* be *C*(*X*). A constraint can be *intravariable*, meaning that it is imposed on a single variable. For example, there may be lower and upper bounds for each variable *x*_*i*_ as *x*_*iL*_ ≤ *x*_*i*_ ≤ *x*_*iU*_, *i* = 1, *k*. The set of such constraints for all *x*_*i*_, *i* = 1, *k*, would then represent a “hyperbox” in a *k*-dimensional space for *x*_*i*_, *i* = 1, *k*. A constraint can be *intervariable*, meaning it is imposed between multiple variables; for example, for specific values of *i* and *i*′, *x*_*ij*_ ≥ *x*_*i*′*j*_, *j* = 1, *n*. In a *k*-dimensional space for *x*_*i*_, *i* = 1, *k*, this constraint indicates that all sample points must be in one side of the hyperplane of *x*_*ij*_ = *x*_*i*′*j*_, *j* = 1, *n*. All the above-mentioned constraints can be elements of *C*(*X*), and they represent an ultimate set of constraints, such that every constraint in *C*(*X*) must be satisfied, and conversely, if every constraint in *C*(*X*) is satisfied, the generated random data is *C*(*X*) valid.

Let *S*_1_ = (*π*_11_,…, *π*_1*μ*_) and *S*_2_ = (*π*_21_,…, *π*_2*ν*_) be two sets of parameters associated with a problem. Let the sets of constraints to be imposed on *S*_1_ and *S*_2_ be *C*(*S*_1_) and *C*(*S*_2_), respectively. Obviously the constraints *C*(*X*), *C*(*S*_1_), and *C*(*S*_2_), or *n* data samples generated under these constraints, must be consistent with each other. For example, if *C*(*X*) says all variables must be nonnegative and some of randomly generated variables under *C*(*S*_2_) are negative, *C*(*X*) and *C*(*S*_2_) are not consistent and the resulting data is not *C*(*X*) valid.

We can consider multidimensional spaces defined on *X*, *S*_1_, and *S*_2_. For *X*, it is a *k*-dimensional space corresponding to *k* variables, *x*_1_,…, *x*_*k*_. For *S*_1_ and *S*_2_, they are *μ*- and *ν*-dimensional spaces, respectively, corresponding to the number of parameters. The set of constraints for each space will specify a certain region (domain) within the space. For example, for *X*, *C*(*X*) will specify a certain domain in the *k*-dimensional space. Randomly generated *n* events for a specific run will be represented as scattered points within the domain. For *S*_1_ and *S*_2_, *C*(*S*_1_) and *C*(*S*_2_), respectively, will specify their domains. For example, for *S*_1_, parameters *π*_11_,…, *π*_1*μ*_ must be confined within the domain that satisfies *C*(*S*_1_).

The major contributions of this article are to

1. point out some potential inconsistencies of random data generation,
2. discuss methods of detecting such inconsistencies,
3. propose techniques of avoiding or correcting such inconsistencies.

We illustrate these concepts on three widely researched problems: a queuing problem, fluid dynamics, and the total tardiness problem. The total tardiness problem is an NP-hard job scheduling problem [2] that “continues to attract significant research interest from both a theoretical and a practical perspective” [3].

We characterize two types of parameters called *data* and *characteristic* parameters for easy reference. The former are often naturally derived parameters directly associated with the random data. The latter are additional parameters introduced to better represent the characteristics of the problem overall, such as the difficulty of the problem. These parameter sets can be corresponded to *S*_1_ and *S*_2_ described earlier.

Section 2 considers simple examples to show how constraint violations may occur among different parameter sets. In Sections 3 and 4, details of the total tardiness problem are discussed in the form of a case study. In particular, Section 3 reviews the total tardiness problem and Section 4 discusses various types of constraints and how their violations can occur. Sections 5–8 discuss various correction algorithms for different types of constraint violations.  Section 9 presents results of a numerical experiment.  Section 10 provides general guidelines for generating stochastic data under constraints and recommends possible further studies. The basic concepts discussed in the problems are applicable to any other problems that employ random data involving parameter sets and constraints.

## 2. Simple Illustrations of Multiple Parameter Sets

We consider two relevant examples in this section—a simple queuing problem [4] and fluid dynamics [5].

### 2.1. Queuing Problem

Customers arrive stochastically at a service station of *m* servers at rate *λ* and are served at rate *μ* for each server (*mμ* represents the service rate at the entire station). These parameters *m*, *λ*, and *μ* can be considered as data parameters since they are directly associated with the randomly generated data. In addition, the traffic intensity *ρ* = *λ*/(*mμ*) is the steady-state fraction of the time in which the server is busy, and characterizes the difficulty of the problem—the higher the *ρ* value, the harder the problem. Thus, the parameter *ρ* can be considered as a characteristic parameter, since it is derived indirectly from data parameters and it is for the purpose of characterizing the problem as a whole.

When constraints are imposed on different parameter sets, constraints on one parameter set must be consistent with constraints on the other parameter set so that randomly generated data are consistent under both parameter sets. Common constraints on *ρ* are 0 ≤ *ρ* ≤ 1; the first nonnegative condition must hold since all the parameters involved are nonnegative. The second condition is assumed since the queue grows infinitely otherwise. One can perform simulation of the queuing problem selecting various values of *m*, *λ*, and *μ*, under certain types of probability distributions (e.g., Poisson and exponential) for arrivals and services. Suppose that we arbitrarily set the ranges of *m*, *λ*, and *μ* as, for example, *m* = [1, *m*_max⁡_], *λ* = [0, *λ*_max⁡_], and *μ* = [0, *μ*_max⁡_], and consider all discrete combinations of (*m*, *λ*, *μ*) in these ranges. Obviously some (*m*, *λ*, *μ*) triplets can violate the 0 ≤ *ρ* ≤ 1 constraints (e.g., triplets with *m* = 1, *λ* > *μ* are violations).

Such constraint violations may be trivial in the above queuing example since the number of parameters is small, and the associated constraints are straightforward. When the number of parameters becomes larger and the associated constraints are more complex or dynamic, however, violations may not be so obvious.

### 2.2. Fluid Dynamics

Many fluid dynamics phenomena are highly nonlinear and present challenging problems both theoretically and experimentally. For example, in the year 2000 a US based mathematical society announced the seven “Millennium Prize Problems.” These are considered some of the world's hardest unsolved problems and a $1 million prize is posted for each question. One problem is in fluid dynamics and is concerned with the Navier-Stokes equation.

Since solving the whole Navier-Stokes equation is very difficult, usually researchers consider special cases under certain assumptions or simplifications. For this purpose, a characteristic parameter called the Reynolds number, *R*, is commonly employed:

(1) $$\begin{array}{c} R=\frac{dvL}{\eta }, \end{array}$$

where *d* is the density, *v* the velocity, *L* a characteristic length scale, and *η* the viscosity. *R* characterizes the flow's likelihood of being turbulent or laminar; the higher the *R* is, the more likely there is to be turbulence. Suppose one wants to study a hard fluid dynamics problem that involves random noise. One would numerically experiment with the flow by generating random data for the noise and by considering various values of *R* and the data parameters of the right-hand side.

This is similar to the scenario discussed in the queuing problem. For example, suppose we consider only discrete values of the parameters. Similar to the queuing problem, upper and lower bounds can be set for each parameter. Constraints on *R*, *R*_min⁡_ ≤ *R* ≤ *R*_max⁡_ may indicate the study is to be performed only for nonturbulent, laminar flows or certain types of turbulent flows. If we consider all combinations of *d*, *v*, *L*, and *η*, some parameter value combinations may not be consistent with the constraints for *R* and the resulting data may be flawed. The types of characteristic and data parameters discussed in this section are common in many disciplines; hence we should be cautious when random data generation is considered.

## 3. The Total Tardiness Problem: A Case Study 

We briefly describe the static total tardiness problem; that is, parameter values do not change dynamically. At time *t* = 0, *n* jobs wait for processing. For the simplest, single machine model, each job is processed by the single machine one at a time. For each problem instance, the *n* jobs have *processing times p*_1_, *p*_2_,…, *p*_*n*_ and *due dates d*_1_, *d*_2_,…, *d*_*n*_, respectively. The objective of the total tardiness problem is to determine the order of jobs to be processed to minimize the *total tardiness*, that is, the total number of days past due dates of tardy jobs (jobs whose completion times exceed their due dates). (Note that although “days” are used here, any other time units such as hours and minutes can be employed depending on a specific application.)

We use the following notations: subscripts: *s*: “preset” or assigned, *e*: “expected,” *a*: “actual,” *L*: lower bound, *U*: upper bound; parameters: *n*: number of jobs in each problem instance; (note: in job scheduling research including tardiness it is customary to call “a problem instance” simply “a problem” and we follow this practice hereafter. A problem is specific values of *n* jobs with associated values of *p*_*i*_s and *d*_*i*_s)

 
*p*: processing time: processing time for each job is denoted by *p*_*i*_, 1 ≤ *i* ≤ *n*;
 
*p*_*L*_, *p*_*U*_: pre-set lower and upper bounds of processing time;
 
$\overset{-}{p}$: average processing time: in most research works, a uniform distribution between *p*_*L*_ and *p*_*U*_ is assumed for processing time *p*_*i*_; in this case, the pre-set average processing time ${\overset{-}{p}}_{s}$ is (*p*_*L*_ + *p*_*U*_)/2; this value may or may not be the same as the expected average processing time ${\overset{-}{p}}_{e}$ for a specific random data generation method and is typically different from the actual average processing time ${\overset{-}{p}}_{a}$ for a specific run for a specific random data generation method; the “expected average” of any variable *x*_*i*_, 1 ≤ *i* ≤ *n*, is computed by ∑_*i*=1_^*n*^ *P*_*i*_ *x*_*i*_, where *P*_*i*_ is the probability of *x*_*i*_.
 
*d*: due date: due date for each job is denoted by *d*_*i*_, 1 ≤ *i* ≤ *n*; other notations for processing time, such as lower and upper bounds, and average for pre-set, expected and actual, also apply for *due date*; in particular, a uniform distribution between *d*_*L*_ and *d*_*U*_ is commonly assumed for due date *d*_*i*_; in this case, the pre-set average due date ${\overset{-}{d}}_{s}$ is (*d*_*L*_ + *d*_*U*_)/2.

Two common parameters, *τ* and *δ*, called the *tardiness factor* and *relative range* of *due dates*, respectively, are employed to characterize a specific tardiness problem. Their definitions and meanings are as follows:

(2) τ=1−d¯s(n·p−s)=1−(dL+dU)(2n·p−s),0≤τ≤1,

(3) $$\begin{array}{c} \delta =\frac{\left(d_{U}-d_{L}\right)}{\left(n\cdot {\overset{-}{p}}_{s}\right)},\;0\leq \delta \leq 1. \end{array}$$

The tardiness factor *τ* represents the average ratio of the number of jobs that do not finish on time over the total number of jobs. The relative range of due dates, *δ*, is a measure for the due date span (*d*_*U*_ − *d*_*L*_) over the total processing time $\left(n\cdot {\overset{-}{p}}_{s}\right)$. In practice, *τ* and *δ* may be pre-set to specific values (e.g., *τ* = 0.8 and *δ* = 0.2) first, and then *d*_*U*_ and *d*_*L*_ may be subsequently determined. This can be achieved by solving the above two equations for *d*_*L*_ and *d*_*U*_ in terms of *τ* and *δ* as follows:

(4) $$\begin{array}{c} d_{L}=\frac{\left(n\cdot {\overset{-}{p}}_{s}\right)\left\{2\left(1-\tau \right)-\delta \right\}}{2}, \end{array}$$

(5) $$\begin{array}{c} d_{U}=\frac{\left(n\cdot {\overset{-}{p}}_{s}\right)\left\{2\left(1-\tau \right)-\delta \right\}}{2}. \end{array}$$

Note that *d*_*L*_ and *d*_*U*_ differ only for the sign of *δ*.

In the total tardiness problem, *d*_*L*_ and *d*_*U*_ are data parameters, while *τ* and *δ* are characteristic parameters [6]. It is common that stochastic data is generated under constraints that are expressed in terms of different parameter sets. In the illustration here, {*d*_*L*_, *d*_*U*_} is the set of data parameters, and {*τ*, *δ*} is the set of characteristic parameters. Each parameter set (e.g., {*τ*, *δ*}) should satisfy the same constraints imposed by the other parameter set (e.g., {*d*_*L*_, *d*_*U*_}); otherwise, the constraints are violated, resulting in inappropriate data.

Extensive research on solution methods for the tardiness problem has been undertaken. One category of these methods is heuristic approaches which include [7–14].

## 4. Common Data Generation Method for the Total Tardiness Problem

Previous works [12–18] studied the performance of their heuristic algorithms by randomly generating test data. Typically, the test data were generated as follows.

For each job *i*, 1 ≤ *i* ≤ *n*, an integer processing time *p*_*i*_ is generated from the uniform distribution (*p*_*L*_, *p*_*U*_); for example, *p*_*L*_ = 1, *p*_*U*_ = 100. Then $n\cdot {\overset{-}{p}}_{a}=\sum_{i=1}^{n}p_{i}$ is computed, and the *τ* and *δ* values are selected from the {0.2,0.4,0.6,0.8,1.0} set. That is, there will be 5 × 5 = 25 pairs of (*τ*, *δ*) as (0.2,0.2), (0.2,0.4),…, (1.0,1.0). Then for each job *i*, an integer due date *d*_*i*_ is generated from the uniform distribution [*d*_*L*_, *d*_*U*_], where $d_{L}=(n\cdot {\overset{-}{p}}_{a})\{2(1-\tau )-\delta \}/2$ and $d_{U}=(n\cdot {\overset{-}{p}}_{a})\{2(1-\tau )+\delta \}/2$. Five problems are generated for each pair of *τ* and *δ* values, yielding a total of 5 × 25 = 125 problems.

There are two obvious data constraints in the total tardiness problem.

1. Nonnegative due date. The due date for every job must be non-negative; that is, *d*_*i*_ ≥ 0, for 1 ≤ *i* ≤ *n*.
2. Due date ≥ processing time for every job. It is reasonable to assume that, no one will accept a job that takes more time to process than its due date. We would be penalized even if we start such a job first. That is, *d*_*i*_ ≥ *p*_*i*_ must hold for 1 ≤ *i* ≤ *n*.

We note that the first constraint is imposed on *individual* variable *d*_*i*_; hence we call it *intravariable* constraint. The second constraint is between two variables (*d*_*i*_, *p*_*i*_); hence we call it *intervariable *constraint. As discussed later, intervariable constraints are typically harder to deal with than intra-variable constraints. We also note that data parameters {*d*_*L*_, *d*_*U*_} and characteristic parameters {*τ*, *δ*} provide *macro-* (i.e., *problem-level*) characterization of the problem. On the other hand, the intra- and intervariable constraints (*d*_*i*_ ≥ 0 and *d*_*i*_ ≥ *p*, resp.) provide *micro-* (i.e., *job-level*) characterization of the problem. These basic concepts of intra- versus inter-variable constraints and macro- versus microparameters help to better define the nature of the problem.

**Figure 1:**
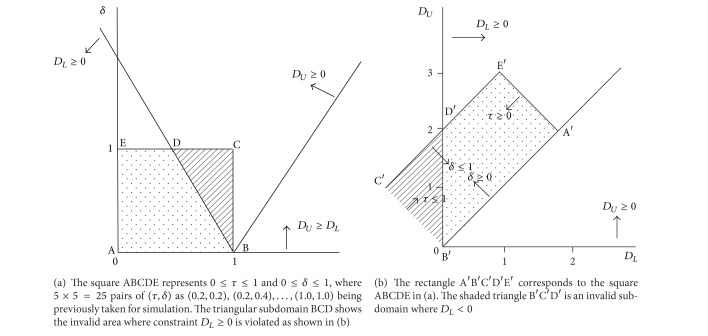
Two-dimensional spaces illustrating invalid subdomains where *D*_*L*_ < 0.

**Figure 2:**
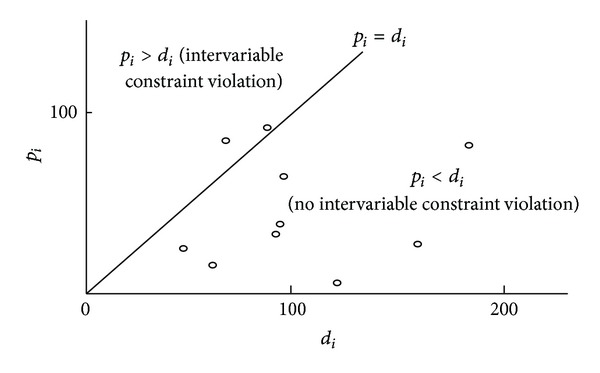
Sample space (*d*_*i*_, *p*_*i*_) for Example 1. Two dots above the *p*_*i*_ = *d*_*i*_ line are inter-variable constraint violations.

The above data generation procedure will yield violations of both intra- and intervariable constraints [19], depending on the values of *τ*, *δ*, *n*, *p*_*L*_, and *p*_*U*_. Why this procedure yields violations can be best understood by examining Figures 1 and 2.  Figure 1 explains why some of the 25 pairs of (*τ*, *δ*) described above lead to intravariable constraint violations (negative due dates). The basic reason is that these (*τ*, *δ*) combinations (in the shaded area of the triangular sub-domain BCD in Figure 1(a)) correspond to negative *d*_*L*_ values (in the shaded area of the triangular sub-domain B′C′D′ in Figure 1(b)). The negative *d*_*L*_ values lead to some of the randomly generated *d*_*i*_ values becoming negative.  Figure 2 illustrates a situation where inter-variable constraint violations occur.

When we compare the shaded/dotted areas of Figures 1(a) and 1(b), we see that there is one-to-one correspondence between every point in Figure 1(a) and every point in Figure 1(b). For example, this is true for the trapezoid ABDE in Figure 1(a) and the trapezoid A′B′D′E′ in Figure 1(b). When two domains (e.g., ABDE and A′B′D′E′) in two different parameter spaces have this one-to-one correspondence property, we say the two domains are *constraint isomorphic* (or simply *isomorphic*). When two domains are isomorphic and constraint violations are identified in one domain, this will help to easily determine constraint violations in the other domain [6]. Similar constraint violation problems are also considered in [20, 21]. We consider correction algorithms for intravariable constraint violations in Section 5 and correction algorithms for intervariable constraint violations in the succeeding sections.

## 5. Correction Algorithms for Intravariable Constraint Violations

We consider various algorithms to avoid or correct intravariable constraint violations.

*Safe-Zone Algorithm*. Use only (*τ*, *δ*) combinations that yield no violations, that is, those in the trapezoid ABDE in Figure 1(a). This is the simplest and easiest approach. When we adopt this algorithm, out of the 25 pairs of (*τ*, *δ*) that were used in the previous work cited earlier, nine combinations would not be considered as they will lead to intravariable constraint violations. These are (*τ*, *δ*) = (0.6,1.0), (0.8,0.6), (0.8,0.8), (0.8,1.0), (1.0,0.2), (1.0,0.4), (1.0,0.6), (1.0,0.8), and (1.0,1.0).

We note that although this approach is the best for its simplicity and was used by many researchers (e.g., [22, 23]), whether it can be legitimately employed is another issue. The problem characteristics, rather than the simplicity of constraint satisfaction, should dictate the selection of parameters. If the problem requires combinations outside the safe zone, we may have to give up this algorithm and rely on other approaches.

*Discard-and-Replace Algorithm*. Whenever negative *d*_*i*_ is generated, either replace it with a constant [24] or simply discard it and continue data generation until the next non-negative *d*_*i*_ is generated [25]. This is a widely used approach for stochastic data generation in general. We must be cautious though, since this discard-and-replace process in general may alter our original data intentions. Here we address three problematic issues relating to (1) the data constraints, (2) the data distribution (uniform, normal, etc.) characteristics, and (3) the characteristic constraints (e.g., (*τ*, *δ*) values). When we employ the discard-and-replace method, obviously (1) the data constraints are satisfied, but (2) the distribution properties and (3) the characteristic values may or may not remain the same. As to the data distribution, if the original distribution is uniform, it is likely that this is preserved (because every *d*_*i*_ is equally likely to be picked over the range [0, *d*_*U*_]). But if the original distribution is nonuniform, by chopping off portion of the range of the random variable (as, for, e.g., *d*_*i*_ < 0) it is likely to change the distribution. In case of the normal distribution, the leftmost portion may be cut off, resulting in a nonsymmetric distribution; that is, it is no longer a normal distribution in the new range.

When we apply the discard-and-replace method to our intravariable constraint violations, the above discussion of data distribution holds. If we assume a uniform distribution as most literature in the total tardiness problem has, the new distribution will remain uniform (again because every *d*_*i*_ is equally likely to be picked over the range [0, *d*_*U*_]). The (*τ*, *δ*) values, however, change because the new *d*_*L*_ = 0. This is a change to a macrocharacterization of the problem. The new (*τ*, *δ*) values can be determined by substituting *d*_*L*_ = 0 into (2) and (3), yielding

(6) $$\begin{array}{c} \tau '=1-\frac{d_{U}}{\left(2n\cdot {\overset{-}{p}}_{s}\right)},\;0\leq \tau \leq 1,\\ \delta '=\frac{d_{U}}{\left(n\cdot {\overset{-}{p}}_{s}\right)},\;0\leq \delta \leq 1. \end{array}$$

Incidentally, substituting these *τ*′ and *δ*′ into (4) gives *d*_*L*_ ≡ 0; that is, (6) and (4) are consistent.

One may wonder about the significance of the discard-and-replace approach in this context. We start with (*τ*, *δ*) value combinations that can cause intra-variable constraint violations and go through the computational process, ending up with the random data with the new (*τ*′, *δ*′) values given in (6). Why not start with the (*τ*′, *δ*′) values from the beginning without discard-and-replace? Yes, this approach should give the same result with much more efficient computing time! In our case, this means using the safe-zone algorithm rather than the discard-and-replace algorithm. We can extend this idea to some other applications. Summarizing, we propose the following. 

*Tip*. Avoid the discard-and-replace method in general. More specifically, consider a two-step process.

**Step 1**

Whenever the discard-and-replace method is employed for random data generation, consider if there are any side effects on the problem characterizations such as (1) the data constraints, (2) the data distribution characteristics, and (3) the associated characteristic constraints. If any side effects exist, then move to Step 2.

**Step 2**

Determine which characterizations need to be preserved or changed. Consider whether the same data can be generated by adjusting some of the characterizations without employing the discard-and-replace method. This approach is likely much more efficient computationally than the discard-and-replace one.

## 6. Analysis of Intervariable Constraint Violations

For any *i*, 1 ≤ *i* ≤ *n*, *d*_*i*_ < *p*_*i*_ is an intervariable constraint violation. This is a microaspect concerned with individual jobs. We first address a macroaspect by considering feasible orderings of the four data parameters, *p*_*L*_, *p*_*U*_, *d*_*L*_, and *d*_*U*_ [6]. The number of all permutations of the four parameters is 4! = 24, but since *p*_*L*_ ≤ *p*_*U*_ and *d*_*L*_ ≤ *d*_*U*_, we are left with six possible permutations. Furthermore, we can assume *p*_*L*_ ≤ *d*_*L*_ and *p*_*U*_ ≤ *d*_*U*_. If *p*_*L*_ > *d*_*L*_, then the shortest due date will be smaller than the shortest processing time, which violates the assumption and constraints. Similarly, *p*_*U*_ > *d*_*U*_ violates the assumption and constraints. These two additional conditions reduce the feasible orderings to the following two:

(7) $$\begin{array}{c} p_{L}\leq p_{U}\leq d_{L}\leq d_{U}, \end{array}$$

(8) $$\begin{array}{c} p_{L}\leq d_{L}\leq p_{U}\leq d_{U}. \end{array}$$

**Case 1 (*p*_*L*_ ≤ *p*_*U*_ ≤ *d*_*L*_ ≤ *d*_*U*_)**

In this case, *d*_*i*_ ≥ *p*_*i*_ for every *i*; hence, inter-variable constraint violations never occur. The condition for which relation (7) holds in terms of *τ* and *δ* can be determined as follows:

(9) $$\begin{array}{c} p_{U}\leq d_{L}=n\left(p_{L}+p_{U}\right)\frac{2\left(1-\tau \right)-\delta }{4}. \end{array}$$

Hence, we have a condition for which an inter-variable constraint violation never occurs as

(10) $$\begin{array}{c} 2\left(1-\tau \right)-\delta \geq \frac{4p_{U}}{n\left(p_{L}+p_{U}\right)}. \end{array}$$

**Case 2 (*p*_*L*_ ≤ *d*_*L*_ ≤ *p*_*U*_ ≤ *d*_*U*_)**

We see that inter-variable constraint violations can occur in this case because the relation *d*_*L*_ ≤ *p*_*U*_ can cause *d*_*i*_ < *p*_*i*_ for some jobs. The following is a simple example, randomly generated following the typical procedure described in Section 4 (Figure 2).

**Example 1**

*n* = 10; *p*_*L*_ = 1, *p*_*U*_ = 100; hence ${\overset{-}{p}}_{s}=50.5$; *τ* = 0.8, *δ* = 0.3; hence *d*_*L*_ = 25.2, *d*_*U*_ = 176.8, and ${\overset{-}{d}}_{s}=101$ (see Table 1).

In this specific example, two jobs violate the inter-variable constraint. As we see below (13), for this particular parameter value combination, the probability of the violation is 0.187; that is, on average 1.87 jobs will have *d*_*i*_ < *p*_*i*_.

When there are values with fractions (e.g., *d*_*L*_ = −25.2), for practical purposes they can be rounded to the nearest integer (e.g., *d*_*L*_ = −25). In the following, we first discuss theoretical analysis of violations and heuristic procedures for how to avoid them [6]. We will primarily use uniform distributions which are employed by most researchers in the tardiness problem.

We must satisfy *p*_*i*_ ≤ *d*_*i*_ for every *i*, but *p*_*i*_ > *d*_*j*_ is perfectly fine for different *i* and *j*. We can show that *τ* and *δ* must satisfy the following three conditions (two conditions in (8)) in terms of *n*, *p*_*L*_, and *p*_*U*_, to have the relation *p*_*L*_ ≤ *d*_*L*_ ≤ *p*_*U*_ ≤ *d*_*U*_:

(11) $$\begin{array}{c} \frac{4p_{U}}{\left\{n\cdot \left(p_{L}+p_{U}\right)\right\}}\geq 2\left(1-\tau \right)-\delta \geq \frac{4p_{L}}{\left\{n\cdot \left(p_{L}+p_{U}\right)\right\}},\\ 2\left(1-\tau \right)+\delta \geq \frac{4p_{U}}{\left\{n\cdot \left(p_{L}+p_{U}\right)\right\}}. \end{array}$$

By adding the last two relations, we also have

(12) $$\begin{array}{c} \tau \leq 1-\frac{1}{n}. \end{array}$$

For example, if *n* = 10, then *τ* needs to be ≤0.9 to satisfy the last two relations.

Let *P*(*d*_*i*_ < *p*_*i*_) be the probability of *d*_*i*_ < *p*_*i*_ for a specific job *i*. This probability is given by the following formula:

(13) P(di<pi)=(pU−dL)32(pU−pL)(dU−dL)(pU−dL+1)

(14) ≈(pU−dL)22(pU−pL)(dU−dL)(when  pU−dL≫1).

The expected number of jobs for which *d*_*i*_ < *p*_*i*_ for a problem of *n* jobs can be determined by *n* × *P*(*d*_*i*_ < *p*_*i*_).

We can use *P*(*d*_*i*_ < *p*_*i*_) to compute other related probabilities. Let *q* = *P*(*d*_*i*_ < *p*_*i*_), for simplicity. The probability of not *d*_*i*_ < *p*_*i*_, that is, *P*(*d*_*i*_ ≥ *p*_*i*_), is 1 − *q*. Out of the total *n* jobs, the probability that exactly *m* jobs are *d*_*i*_ < *p*_*i*_ is

(15) P  (m  out  of  n  jobs  are  di<pi)=Cmnqm(1−q)n−m,

where *q* = *P*(*d*_*i*_ < *p*_*i*_) and _*n*_ *C*_*m*_ is the number of combinations for selecting *m* objects out of *n* objects at a time. This probability distribution for *m* = 0 to *n* is the binomial distribution. In particular, the probability that at least one job has a violation is 1 − *P*  (*m* = 0  jobs  are  *d*_*i*_ < *p*_*i*_) = 1 − (1 − *q*)^*n*^.

## 7. Removing Intervariable Constraint Violations by Simple Approaches

Without loss of generality, let us assume that we generate *p*_*i*_'s first, followed by *d*_*i*_'s, as suggested by previous research. Suppose that *p*_1_ = 15 and *p*_2_ = 95. If *d*_1_ = 45 and *d*_2_ = 125, there are no violations, but if these *d*_*i*_'s are swapped, a violation occurs. That is, we must satisfy *p*_*i*_ ≤ *d*_*i*_ for every *i*, but we do not know the specific values of *p*_*i*_'s and *d*_*i*_'s until they are randomly generated. How to efficiently generate stochastic data that does not violate intervariable constraints is a challenging problem.

### 7.1. Discard-and-Replace Methods

There can be different versions of this classic approach depending on how one picks a new data element.

*Discard-and-Replace with Next Random Valid Values*. This is the most common version of discard-and-replace methods in general. For the tardiness problem, “if a negative processing time value is generated during the simulations, it is simply *ignored* and generated again” [25]. When *d*_*i*_ < *p*_*i*_ we would continue to generate the next random *d*_*i*_, until it satisfies *d*_*i*_ ≥ *p*_*i*_. A side effect of this approach is that it will skew the due date distribution to a higher range. The resulting (*p*_*i*_, *d*_*i*_) distribution will remain stochastic (even though *d*_*i*_'s skewed upward). The following is an illustrative example.

**Example 2 (same as Example 1, except here we discard and replace with random valid values)**

*n* = 10; *p*_*L*_ = 1, *p*_*U*_ = 100; hence ${\overset{-}{p}}_{s}=50.5$; *τ* = 0.8, *δ* = 0.3; hence *d*_*L*_ = 25.2, *d*_*U*_ = 176.8, and ${\overset{-}{d}}_{s}=101$.

Actual values are *d*_*L*,*a*_ = 58, ${\bar{p}}_{a}=104.5$, *τ*_*a*_ = 0.79, and *δ*_*a*_ = 0.23 (see Table 2).

This example is exactly the same as Example 1 except that *d*_*i*_ is replaced with the next *d*_*i*_ whenever a violation *d*_*i*_ < *p*_*i*_ occurs. The pre-set due date average ${\overset{-}{d}}_{s}$ is 101. The actual due date average ${\overset{-}{d}}_{a}$ for Example 1 is 99.2, while that of Example 2 is 104.5, showing, expectedly, an overall increase of *d*_*i*_'s.

We can show that the overall expected *d*_*L*,*e*_ is as follows:

(16) $$\begin{array}{c} d_{L,e}=\frac{\left\{p_{U}\left(p_{U}+1\right)+\left(d_{L}-2p_{L}+1\right)\right\}}{2\left(p_{U}-p_{L}+1\right)}. \end{array}$$

We note that *d*_*L*,*e*_ is the expected value, not the actual value; the actual value is bounded from below by *d*_*L*_. Similarly, the overall expected average due date, ${\overset{-}{d}}_{e}$, is given by

(17) d¯e={pU(pU+1)+dL(dL−2pL+1)+2dU(pU−pL+1)}  ×(4(pU−pL+1))−1.

Since the new distributions are skewed, we are not precisely dealing with *τ* and *δ* as they were specified originally. But, it is most reasonable to define the effective *τ*_*e*_ and *δ*_*e*_ by substituting ${\overset{-}{d}}_{s}$ and *d*_*L*_ in the original definitions of *τ* and *δ* in (2) and (3) with their effective counterparts *τ*_*e*_ and *δ*_*e*_. That is,

(18) $$\begin{array}{c} \tau_{e}=1-\frac{{\overset{-}{d}}_{e}}{\left(n\cdot {\bar{p}}_{s}\right)}=1-\frac{\left(d_{L,e}+d_{U}\right)}{\left(2n\cdot {\bar{p}}_{s}\right)},\\ \delta_{e}=\frac{\left(d_{U}-d_{L,e}\right)}{\left(n\cdot {\bar{p}}_{s}\right)}. \end{array}$$

In Example 2, these effective values are *d*_*L*,*e*_ = 53.6, ${\overset{-}{d}}_{e}=115.2$, *τ*_*e*_ = 0.772, and *δ*_*e*_ = 0.244. We note that (16)–(18) can also be expressed in terms of *n*, *p*_*L*_, *p*_*U*_, *τ*, and *δ*, by using (4) and (5).

As an alternative version of discard-and-replace, one can set *d*_*i*_ = *p*_*i*_ when the generated values are such that *d*_*i*_ < *p*_*i*_. This version of replacing *d*_*i*_ < *p*_*i*_ with *d*_*i*_ = *p*_*i*_ will have two shortcomings. First, the resulting due date distribution will be skewed toward a higher range, as in the previous version, since due dates with *d*_*i*_ < *p*_*i*_ are replaced with higher values of *p*_*i*_. Second, the resulting (*p*_*i*_, *d*_*i*_) distribution will be less stochastic than the previous version since all the jobs with replaced *d*_*i*_ will have exactly the same due dates as their processing times.

### 7.2. Augmented Probability Distributions

In the discard-and-replace method discussed in the previous subsection, we encountered violations of *d*_*i*_ < *p*_*i*_, and replacing *d*_*i*_ with a larger *d*_*i*_ caused distortion of the underlying distribution. Here, we ask whether there are any guaranteed methods in which violations never occur. We can, for example, employ a *d*_*i*_-generation function as follows:

(19) $$\begin{array}{c} d_{i}=p_{i}+h_{i}, \end{array}$$

where *h*_*i*_ is some random function which is *h*_*i*_ ≥ 0. In this way, not only *d*_*i*_ is guaranteed to be ≥*p*_*i*_, but also lower *d*_*i*_ tends to be assigned to lower *p*_*i*_ and higher *d*_*i*_ to higher *p*_*i*_ [26]. Variations of (19) include *d*_*i*_ = *α*_*i*_ *p*_*i*_, where *α*_*i*_ ≥ 1, and a combination of (19) and *d*_*i*_ = *α*_*i*_ *p*_*i*_ as *d*_*i*_ = *α*_*i*_ *p*_*i*_ + *h*_*i*_.

We must, however, be cautious in employing such methods. For example, if we select uniform distributions for *p*_*i*_ and *h*_*i*_ in (19), *d*_*i*_ will not be uniform any more (its probability density function will be trapezoidal). How to reasonably define *τ* and *δ* in such a situation is another question. In short, we need careful consideration before employing these methods.

## 8. A Neighborhood Expanding Data-Interchanging Heuristic

### 8.1. General Description

The method discussed in this section is a heuristic for reducing the impact of constraint violations on the generated data. The basic idea of this method should be applicable to many types of problems. 

**Figure 3:**
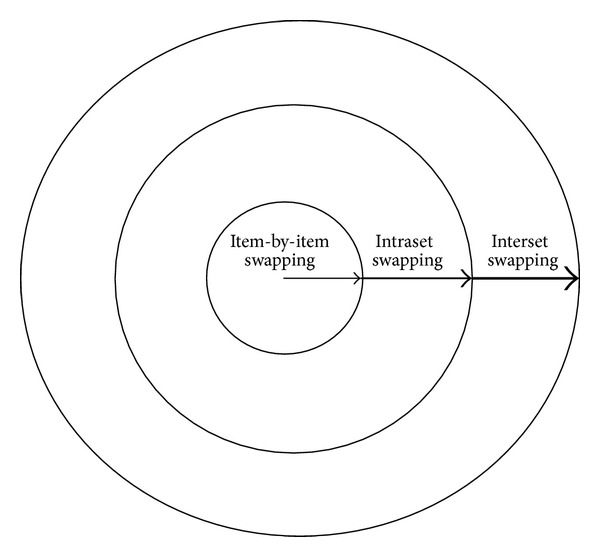
A schematic representation of the neighborhood expanding data-interchanging heuristic. As the neighborhood expands, the probability of finding pairable swaps increases (indicated by the thickness of the arrow).

*General Idea*. We study randomly generating values of variable *x*_*i*_. A set of these values may contain *n* values for *x*_*i*_, *i* = 1, *n*. Further, we can extend the size of the data as a group of multiple sets and a group of groups of sets and so forth. We consider the neighborhood of these data. The most local neighborhood of *x*_*i*_ can be the neighboring data elements of *x*_*i*_ as, for example, *x*_*i*−1_, *x*_*i*_, *x*_*i*+1_. When the neighborhood coverage of data elements is extended to the entire set or a group of sets and so forth, the scope of the neighborhood will be more “global.” We perform data interchanging starting from the most local neighborhood level to resolve violations. If they are not resolved, we extend the neighborhood toward a more global level, until all violations are resolved (Figure 3). Hence, we use the following steps.

- Item-by-item swapping at the most local level: when a violation is found for a specific data item, find another data item such that when these two data items are swapped the violation is resolved.
- Intraset swapping: when the above item-by-item swapping does not work, consider the entire data set in which the data item is an element. Swap any data items (elements) within the set so that violations can be removed.
- Interset swapping: when the above intra-set swapping does not work, include neighboring data sets to the above data set, and try to resolve violations by taking into account all of the data items in all the data sets under consideration. Start with an adjacent data set, expanding toward the entire collection of data sets until violations are resolved.
- If either the inter-set swapping does not work or there are no other data sets to include, discard and replace some data item(s) or data set(s). Hopefully, the chances of performing this last step are very small.

### 8.2. An Illustration Using the Total Tardiness Problem 

#### 8.2.1. Preliminaries

The heuristic here is a special case of the above basic idea of the neighborhood expanding data-interchanging method, where “data item” and “data set” are replaced by “job” and “problem,” respectively. In the implementation of the heuristic, we skip the most local, item-by-item swapping, described in the above general outline of the method, since it does not appear particularly effective for the inter-variable violation problem. For some other types of violations, this step may be useful. Before describing the heuristic, we introduce a term and a theorem.

**Definition 3**

Processing times and due dates of a problem are *pairable* if there is at least one permutation of *p*_*i*_'s and at least one permutation of *d*_*i*_'s that satisfy *p*_*i*_ ≤ *d*_*i*_ for every *i* = 1 to *n*; in this case, we say that the problem is pairable. In other words, if a problem is pairable, we can make an invalid problem internally valid by rearranging *p*_*i*_'s and *d*_*i*_'s; otherwise, it is impossible to make the problem internally valid, no matter how we shuffle *p*_*i*_'s and *d*_*i*_'s.

**Theorem 4**

Sort all *p*_*i*_'s and *d*_*i*_'s in a problem, so that *p*_1_ ≤ *p*_2_ ≤ ⋯ and *d*_1_ ≤ *d*_2_ ≤ ⋯. A necessary and sufficient condition for a problem to be pairable is *p*_*i*_ ≤ *d*_*i*_ for every *i* = 1 to *n*.

**Proof**

If *p*_*i*_ ≤ *d*_*i*_ for every *i* = 1 to *n* for sorted sequences of *p*_*i*_'s and *d*_*i*_'s, the set of the sorted sequences is an internally valid problem. Therefore, we can make at least one (and possibly many more) internally valid problem(s). Hence, the condition is sufficient. Conversely, suppose that *p*_*i*_ > *d*_*i*_ for some *i*. Then this *p*_*i*_ must be paired with another *d*_*j*_, *j* > *i*. This leaves fewer *d*'s than *p*'s for pairing (the pigeon-hole principle), which means that pairing all the remaining *p*'s and *d*'s is impossible. Thus, the condition is necessary.

*Data-Interchanging Heuristic*

 Step 1 (intraproblem swapping)
 Sort all *p*_*i*_'s and *d*_*i*_'s (i.e., *p*_1_ ≤ *p*_2_ ≤ ⋯ and *d*_1_ ≤ *d*_2_ ≤ ⋯).
 For *i* = *n* step −1 down to 1 do
   For *p*_*i*_, find the smallest *d*_min⁡_ such that *d*_min⁡_ ≥ *p*_*i*_.
   Randomly select *d*_*j*_ in *d*_min⁡_ to *d*_*i*_.
   Pair (*p*_*i*_, *d*_*j*_) and output it as a valid pair.
   Rearrange *d*_*k*_ by *d*_*k*_ ← *d*_*k*+1_ for *k* = *j* to *i* − 1.
   Enddo.
 Restore the original (presorting) order of *p*_*i*_'s for the generated *n* pairs of (*p*_*i*_, *d*_*i*_); that is, *p*_*i*_'s in new pairs of (*p*_*i*_, *d*_*i*_) appear in the same order as originally generated at random. (so that *p*_*i*_'s are not in any particular sequence such as being sorted).
 Step 2 (interproblem swapping). When Step 1 does not work, include gradually increasing number of neighboring problems to the above problem. We may start with combining two problems, the above problem and the succeeding problem, having a total of 2*n* jobs, and apply Step 1 to this 2*n*-jobs problem. When 2*n* jobs are successfully paired, restore the original (pre-sorting) orders of *p*_*i*_'s in each problem. If this does not work, include three problems and so on, until the entire problem set is used. Apply Step 1 to each *kn*-jobs problem, where *k* = 2 to number of problems.
 Step 3. If Step 2 does not work, or there is no other problem in Step 2, discard and replace some *d*_*i*_'s, jobs, or problems. Of course, such discard-and-replace process will distort the original data characteristics, like other methods, as discussed previously. Our experiments, as discussed in Section 10, show that the chances of performing Step 3 are extremely small.

#### 8.2.2. Additional Notes on the Data-Interchanging Heuristic

*Item-by-Item Interchanging*. In the above algorithm, although we skipped the most local data interchanging described in the general method, we briefly discuss it here to illustrate how the concept can be applied. 

*Job-by-Job Swapping for the Total Tardiness Problem*. When a violation is found for a specific job *i*, find another job *j* such that *d*_*i*_ ≥ *p*_*j*_ and *d*_*j*_ ≥ *p*_*i*_, and swap *d*_*i*_ and *d*_*j*_ (or *p*_*i*_ and *p*_*j*_). A choice of swapping (*d*_*i*_ and *d*_*j*_) or (*p*_*i*_ and *p*_*j*_) can also be made randomly to avoid, for example, larger *d*_*i*_ tending to appear earlier.

We note that this procedure does not accomplish the same result as Step 1 in the heuristic. Consider the following example.

**Example 5**

(see Table 3) Since job 1 is a violation, we search for *d*_*i*_ ≥ *p*_*j*_ and *d*_*j*_ ≥ *p*_*i*_, but this search fails even though the problem is pairable. We may call such a situation a “*three-way deadlock*.” There can be extensions of this, as *four-way,…, n-way deadlocks*.

*Effect of Step 2 on Data Characteristics.* In Step 2 of the heuristic, we combine two, three, and more problems as needed to come up with valid sequences of *p*_*i*_'s and *d*_*i*_'s. We might wonder whether in effect this process changes the problem size from *n* to 2*n*, 3*n*, and so on. If so, the process would affect the values of *τ* and *δ*, since they depend on the problem size. However, this is not the case. For pairing purposes, we scramble *p*_*i*_'s and *d*_*i*_'s of multiple problems. But after pairing is complete, the original order of *p*_*i*_'s in each problem is restored and the problem size remains the same as *n*.

## 9. Experimental Results

The data-interchanging heuristic was implemented and tested with *n* = 8, 16, 32, 64, and 128; *p*_*L*_ = 1, *p*_*U*_ = 100; hence ${\overset{-}{p}}_{s}=50.5$; *τ* = 0.8 and *δ* = 0.3. *d*_*L*_ and *d*_*U*_ can be determined by using (4) and (5), respectively, as *d*_*L*_ = 2.5*n* and *d*_*U*_ = 17.7*n*. For each problem size *n*, data for *w* = 100 problems of the given size were generated and the violations in the generated data as well as after each of the three steps of the proposed algorithm were recorded (see Table 4). For example, generating 100 problems of size 8 each, the generated data had a total of 365 violations. Using Step 1 of the proposed heuristic, 91 violations remained—a 75% reduction in the number of violations in the generated data. When Step 2 of the heuristic is used, only 25 violations remained—an impressive 93% reduction in the number of violations in the generated data. When Step 3 of the algorithm is used, there was a 100% reduction in the violations in the generated data. Similar performance is observed for other values of *n*. For example, for *n* = 16 and 32, a 100% reduction in the violations was achieved after Step 2 and no further steps were required. While there is no guarantee that such a reduction is expected for every data in general, the result is indicative for effectiveness of the algorithm. Notice that as *n* increases, the probability of having a violation decreases, because *p*_*L*_ and *p*_*U*_ remain constant while *d*_*L*_ and *d*_*U*_ increase with *n*. This is why no violations were encountered for *n* = 64 and 132.

## 10. Conclusions

In this paper, we discussed how implicit constraints were overlooked in some previous practices for generating data to simulate the total tardiness problem. This may not be an isolated case and may extend to other practical approaches involving generation of random data under constraints. When there are possible data violations, analytical approaches such as the one demonstrated in this paper (e.g., Section 4) should be helpful. Heuristics, such as the local-and-global data-interchanging heuristic discussed in this paper, may be used, depending on the nature of the application problems and the types of data violations.

The following are some general guidelines for generating stochastic data under constraints.

1. Carefully examine the problem to see whether there are certain constraints that must be satisfied (e.g., due dates must be non-negative and each due date must be not less than the processing time in the total tardiness problem).
2. Check the procedure of generating stochastic data to determine whether it possibly yields invalid data which is in violation of a constraint. (In the total tardiness problem, by glancing at (3), we see that *d*_*L*_ can be negative for certain values of *τ* and *δ*, thus possibly yielding negative due dates. Also, by looking at (4), we see that *d*_*U*_ can be less than *p*_*L*_, thus possibly generating a job whose due date is less than its processing time.)
3. We need to pay special attention when “characteristic parameters” (e.g., *τ* and *δ*) are introduced. These characteristic parameters are important metrics for the problem to be solved, but they are often abstract and only indirectly represent the original characteristics of the data. This may lead to a common error of focusing primarily on the characteristic parameters and forgetting the nature of the original data.
4. If there may be possible violations, we can theoretically analyze the conditions for which the violations occur. For certain cases, this analysis may lead to a simple revised procedure that guarantees no violations, or a set of parameter values that avoid violations.
5. Whenever the discard-and-replace method is employed, we must consider the resulting effect in terms of the problem characterizations such as (1) the constraints, (2) the data distribution, and (3) the associated characteristics. Determine which characterizations need to be preserved or changed. Consider whether the same data can be generated by adjusting some of the characterizations without employing the time-consuming discard-and-replace method. This approach is likely much more efficient computationally than discard-and-replace.
6. For certain problems, Steps (3) and (4) above may not result in a sufficient method. That is, there is no simple procedure that guarantees no violations, or a set of parameter values that completely avoids violations. In such cases, one may attempt to develop a new data generation procedure that satisfies validity criteria such as the following.
  - No invalid data has been generated.
  - The associated characteristics of data generated are as close as possible to the intended original data (e.g., average and expected values of certain entities), unless the original characteristics themselves are violations.
  - The procedure is computationally simple and efficient.
7. Developing such a procedure satisfying all the above criteria, however, may not be trivial. Often we may find conflicting trade-offs among the various criteria. Usually criterion (a) is the highest priority. Unless we produce massive data, criteria (c) may not be a high priority in comparison with the other criteria, due to the high speed of today's computers. In certain cases, heuristics that are not perfect but practically good enough methods may be used.

Further studies can include the following.

1. Other problems: we employed the total tardiness problem and the two simple examples of Section 2 to illustrate the core of this article, that is, constraint consistency among different parameter sets. Other problems in different domains for various application types can be considered.
2. Nonuniform distributions of random variables: in this article, we primarily focused on uniform distributions since they have been most commonly employed in the total tardiness problem. However, other distributions can also be considered, especially for other problems.
3. Higher number of data and characteristic parameters: for the total tardiness problem, the number of data parameters is two and the number of characteristic parameters is also two. For the two simple examples discussed in Section 2, the number of characteristic parameter is one, and there are several data parameters. We can consider higher number (e.g., three and three, or generally *M* and *N*) for these parameters.
4. More general mapping between data and characteristic parameters: for the total tardiness problem, the mapping is one to one. Other cases, such as many to one, may be considered.

## Figures and Tables

**Table 1:** 

Job no., *i*	1	2	3	4	5	6	7	8	9	10
Proc. time, *p*_*i*_	92	41	10	21	37	86	85	66	25	37
Due date, *d*_*i*_	90	95	116	64	151	171	66	97	49	93
Is *d*_*i*_ < *p*_*i*_?	y						y			

**Table 2:** 

Job no., *i*	1	2	3	4	5	6	7	8	9	10
Proc. time, *p*_*i*_	92	41	10	21	37	86	85	66	25	37
Due date, *d*_*i*_	95	116	64	151	171	97	93	69	131	58

**Table 3:** 

Job no., *i*	1	2	3
Processing time, *p*_*i*_	10	8	5
Due date, *d*_*i*_	7	11	9

**Table 4:** Number of violations in generated data and after each of the steps of the proposed heuristic.

*n*	In generated data	After Step 1	After Step 2	After Step 3
8	365	91	25	0
16	131	86	0	0
32	14	14	0	0
64	0	0	0	0
128	0	0	0	0
